# Impact of social environment on sexual behaviors of adolescent girls in 12 sub-Saharan African countries: a cross-sectional study

**DOI:** 10.1186/s12978-022-01448-0

**Published:** 2022-06-16

**Authors:** Sandra A. Darfour-Oduro, Diana S. Grigsby-Toussaint

**Affiliations:** 1 Department of Epidemiology and Disease Control, School of Public Health, University of Ghana, Accra, Ghana; 2 Department of Behavioral and Social Sciences, Brown University School of Public Health, Providence, RI USA; 3 Department of Epidemiology, Brown University School of Public Health, Providence, RI USA

**Keywords:** Adolescent girls, Social environment, Sexual debut, Sexual behaviors, Sub-Saharan Africa, Parental monitoring

## Abstract

**Background:**

Early sexual debut among adolescent girls may result in teenage pregnancy and sexually transmitted diseases. In sub-Saharan Africa (SSA), pregnancy among adolescent girls may adversely impact the continuation of their education, general health status, and birth outcomes. However, few cross-national studies have examined the role that the social environment plays in adolescent girls' sexual behaviors in SSA. In this study, we explored adolescent girls' social environment and the impact on their sexual behaviors..

**Methods:**

The country selection was based on availability of Global School-Based Student Health Survey (GSHS) national data (2003–2015). The total analytic sample was 22,067 adolescent girls from 12 countries in SSA. Descriptive statistics were generated to determine the characteristics of adolescent girls and independent samples t-test analysis were performed to determine whether there were differences between the social environment and age of sexual debut and sexual partners. Logistic regression models were used to determine the association between adolescent girls’ social environment and sexual debut.

**Results:**

The study results showed variations across the 12 countries. Almost one in five (19.9%) adolescent girls reported to have ever engaged in sexual intercourse. Their mean age of sexual debut was 13.21 (13.04–13.37) years and mean number of sexual partners was 2.19 (2.08–2.29). We found that adolescent girls who reported not being connected with their parents were more likely to debut sex (aOR = 1.32, 95% CI, 1.14–1.53, p < 0.000). Parental monitoring was significantly associated with sexual debut but after controlling for the confounding variables (age, class grade and drug use), the association was no longer significantly positively associated. Adolescent girls who felt supported by their peers had a significantly higher number of sexual partners than those who did not feel supported by their peers.

**Conclusion:**

The social environment of adolescent girls plays a very important role in sexual debut, age of sexual debut, and the number of sexual partners. Sexual health policies targeting adolescent girls are likely to achieve positive impacts if they focus on improving parental connectedness and peer support.

## Plain language summary

Adolescent girls in sub-Saharan Africa are at risk of dropping out of school when they get pregnant. There are also several health risks associated with adolescent pregnancies. This study explored factors in the adolescent girl’s social environment which could impact their sexual behaviors. We examined data from adolescent girls in 12 sub-Saharan African Countries, namely, Kenya, Tanzania, Uganda, Ghana, Mauritania, Senegal, Swaziland, Zambia, Botswana, Namibia, Seychelles and Mozambique. After analyzing the data, we found that adolescent girls who reported not being close with their parents were more likely to debut sex. Adolescent girls who felt supported by their friends had a higher number of sexual partners. Thus, support from parents and peers play a vital role in the sexual behaviors of adolescent girls.

## Background

A World Health Organization (WHO) [1] report indicates that a quarter of the world’s population is made up of people between the ages of 10 and 24 years, and about 90% live in low and middle-income countries (LMICs) where fertility is high [2]. Notably, half of the population in sub-Saharan Africa is younger than 18 years and is the fastest growing region in the world [3]. According to UNICEF [4] and WHO[5], this increase in adolescent population growth coincides with a reduction in infectious disease, malnutrition and infant and early childhood mortality. This reduction has shifted the attention to other health issues such as sexual and reproductive health, which has become very important during adolescence.

According to Sawyer et al. [6] a focus on adolescence is central to the success of many public health agendas. For example, efforts made towards achieving the Millennium Development Goal to reduce child and maternal mortality and human immunodeficiency virus /acquired immunodeficiency diseases (HIV/AIDS) [6] have had a positive impact on adolescent health in South-East Asia, Eastern Mediterranean and the African Regions [7]. Notwithstanding this improvement, maternal mortality is the second highest cause of death globally among girls between the ages of 15 and 19 years [7].

Compared to the rest of the world, young women in sub-Saharan Africa (SSA), face a double burden of unplanned pregnancy and HIV risk [8]. Early initiation of sex (before the age of 18) increases the risk of unintended pregnancy [9], creating a major social problem and a public health challenge [9–11] among adolescent girls. Moreover, childbearing at an early age reduces access to education and employment opportunities that these adolescent mothers might have had [12].

Sub-Saharan Africa has the highest level of adolescent fertility in the world, which significantly contributes to the region’s lifetime average of 5.1 births per woman [12]. Pregnancy at an early age is associated with adverse health outcomes for both mother and child [9], including increased risks for low birth weight, preterm delivery, eclampsia and puerperal endometritis [13].

According to Dimbuene and Defo [14] one of the most influential factors on youth sexual behaviors is the family environment, but little has been done to understand how it impacts adolescent sexual health in sub-Saharan Africa. A study by Beguy et al. [9] found that young women who do not live with their parents are more likely to initiate sex. In Nairobi, Kenya, adolescent girls were less likely to have ever had sex, to have had an unwanted pregnancy or to have been recently sexually active if they live with their fathers [15]. In Ghana, high parental monitoring was negatively associated with adolescent girls being sexually active [16].

A stronger parent–child relationship and higher levels of parental control decreased the risk of premarital intercourse [14]. Diclemente et al. [17] also found that adolescents who perceived less parental monitoring were more likely to test positive for a sexually transmitted disease {odds ratio; OR = 1.7}, not use condoms at the last sexual intercourse {OR = 1.7}, to have multiple sexual partners in the past 6 months {OR = 1.7} and have a new sex partner in the past 30 days {OR = 3.0}. Studies have also found peer influence on adolescent sexual debut [18, 19]. For example, longitudinal studies have found that adolescents are more likely to postpone sexual debut if they have friends who favored postponing sexual intercourse [20–22]. A qualitative study conducted in Ghana also found that adolescent girls, who had not had sex before, were often teased by their peers [23].

The study utilizes the conceptual model of the relationship between social networks and social support on health proposed by Heaney & Israel [24]. This model shows social networks and social support as the starting point or initiator of a causal flow towards health outcome. Social network refers to the web of social relationships that surround individuals, and social support is one of the important functions of social relationships [24]. The model clearly shows that social networks and social support may influence behavioral risk factors and preventive health practices. The study utilized this model to understand better the potential impact of adolescent social environment (parental monitoring, parental and peer connectedness) on their sexual behaviors and sexual health risk.

Thus, developing positive child-parent relationships characterized by connectedness and monitoring, in addition to perceived peer support could lead to lower risky sexual and reproductive health behaviors. To date, there have been few cross-national studies in sub-Saharan Africa exploring the social environment of adolescent girls and the impact on their sexual health. Consequently, the aim of this study is to examine the impact of parental attitudes and peer influence on the sexual debut of adolescent girls in sub-Saharan Africa. The study objectives are, (a) to estimate the prevalence rate of initiation of sexual relationship among adolescent girls and to determine the mean age of sexual debut, and mean number of sexual partners; (b) to estimate the levels of parental monitoring, parental connectedness and peer support among adolescent girls and (c) examine the association between adolescent girls' social environment and sexual debut, age of sexual debut and number of sexual partners.

We hypothesized that adolescent girls who were monitored by and felt connected to their parents and felt supported by their peers would have lower odds of experiencing sexual debut.

## Methods

### Research context

The Global School-based Student Health Survey (GSHS) was the primary data source for this study. The GSHS is a standardized survey developed by the WHO in collaboration with the United Nations International Children’s Emergency Fund (UNICEF), the United Nations Educational, Scientific and Cultural Organization (UNESCO), and the United Nations Program on HIV/AIDS, with technical assistance provided by the Centers for Disease Control and Prevention (CDC). The GSHS is a collaborative surveillance project designed to help countries measure and assess health behaviors and protective factors among students between the ages of 13 and 17 years. The GSHS has ten key questionnaire modules and two of the modules address protective factors and adolescent sexual behaviors [25].

**Fig. 1:**
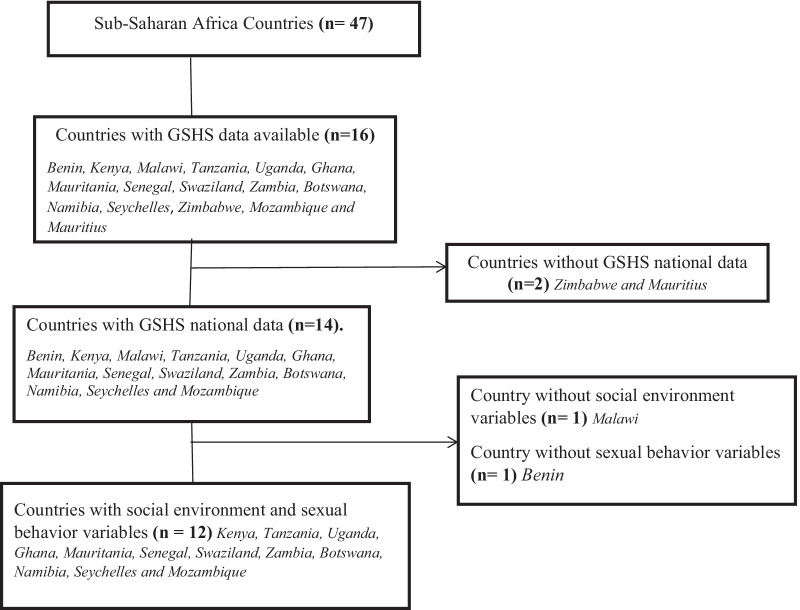
Selection process for SSA countries included in the study using available GSHS data (2003–2015)

The country selection was based on publicly available GSHS national data and data availability on adolescent girls’ social environment and sexual behaviors in sub-Saharan Africa from currently available data (2003–2015) [26]. The total number of sub-Saharan African countries included in the study was 12 with 12,067 adolescent girls (Fig. 1). The Countries were Kenya, Tanzania, Uganda, Ghana, Mauritania, Senegal, Swaziland, Zambia, Botswana, Namibia, Seychelles and Mozambique. Adolescent girls in these 12 countries face similar health and social challenges when they get pregnant at an early age. According to the UN, having children at an early age reduces access to education and employment opportunities [12]. There is also the increased risk for low birth weight, preterm delivery, eclampsia and puerperal endometritis [13]. The study did not require approval by the institutional review board.

### Study design

This study utilizes the secondary cross-sectional study design, drawing on primary data collected from the Global School-based Student Health Survey (GSHS).

### Study participants

The study participants were 12,067 school going adolescent girls from 12 sub-Saharan African Countries.

### Measurement and variables

The study utilized the conceptual model of the relationship between social networks and social support on health proposed by Heaney and Israel [24]. Measures of the social environment used were parental monitoring, parental connectedness, and perceived peer support. Sexual behavior measures were sexual debut, age at first sexual intercourse, condom use and number of sexual partners. The survey measures used were based on the following GSHS questions [27]:

**Table:** 

Type of variables	Name of variables	Questions and responses	Coding
Social environment	Parental monitoring	During the past 30 days, how often did your parents or guardians really know what you were doing with your free time? a) Never b) Rarely c) Sometimes d) Most of the time e) Always	1 = monitored (most of the time, always and sometimes) 2 = not monitored (rarely and never)
	Parental connectedness	During the past 30 days, how often did your parents or guardians understand your problems and worries? a) Never b) Rarely c) Sometimes d) Most of the time e) Always	1 = connected (most of the time, always and sometimes) 2 = not connected (rarely and never)
	Perceived peer support	During the past 30 days, how often were most of the students in your school kind and helpful? a) Never b) Rarely c) Sometimes d) Most of the time e) Always	1 = supported (most of the time, always and sometimes) 2 = not supported (rarely and never)
Sexual Behaviors	Sexual Debut	Have you ever had sexual intercourse? a) Yes b) No	1 = Yes 2 = No
	Age of Sexual intercourse	How old were you when you had sexual intercourse for the first time? a) I have never had sexual intercourse b) 11 years old or younger c) 12 years old d)13 years old e) 14 years old f) 15 years old g) 16 years old or older	1 = 11 years 2 = 12 years 3 = 13 years 4 = 14 years 5 = 15 years 6 = 16 years
	Number of Sexual partners	During your life, with how many people have you had sexual intercourse? a) I have never had sexual intercourse b) 1 person c) 2 people d) 3 people e) 4people f) 5 people g) 6 or more people	1 = 1 person 2 = 2people 3 = 3 people 4 = 4 people 5 = 5 people 6 = 6 people
	Condom use	The last time you had sexual intercourse; did you or your partner use a condom? a) I have never had sexual intercourse b) Yes c) No	1 = Yes 2 = No

Adolescent girls who answered “I have never had sexual intercourse” were excluded in the data analysis for 1) age of sexual intercourse, 2) number of sexual partners and 3) condom use

### Data analysis

The data was analyzed using SPSS version 21 software (SPSS, Inc., Chicago, IL 2018). The GSHS uses the complex sampling design and this was accounted for in the data analysis. The weighting process was used. The weighting process used adds weights, stratum and primary sampling unit (PSU) to every student record in the GSHS data file to reflect the weighting process and the two-staged sampling design. The weight variable allows generalizability of the GSHS results to the entire students’ population whereas the stratum and PSU account for the two-stage sample design used for the GSHS. Specifically, the stratum reflects the GSHS sampling process at the first level which is done in schools and the PSU reflects the second level of the GSHS sampling process conducted in classrooms [28].

Descriptive statistics were generated to determine the number and percentage of adolescent girls who were monitored and felt connected with their parents as well as girls who reported being supported by their peers. The number and percentage of adolescent girls who had initiated sex and used condoms were also analyzed. The mean age of sexual debut and the mean number of sexual partners were also determined. Results were presented for each of the 12 countries and then for all countries.

An independent samples t-test analysis was performed to determine whether there were differences between (i) the mean age of sexual debut based on parental monitoring, parental connectedness, and perceived peer support and (ii) the mean number of sexual partners based on parental monitoring, parental connectedness, and perceived peer support. Data analysis was performed for each country and then for the pooled data set for 12 countries.

Logistic regression models were used to measure the association between each of the social environment components (i.e., parental monitoring, parental connectedness, and perceived peer support) and sexual debut.. Crude odds ratios (OR) of these associations were generated, and then adjusted for age, class grade and drug use for each country. 'We pooled data for 12 countries to determine the association between parental attitudes and perceived peer support on adolescent girls’ sexual behaviors. The crude ORs and adjusted ORs were generated for this analysis.

## Results

The study results showed that more than 50% of adolescent girls reported being monitored by their parents or guardians, felt connected with their parents, and perceived having supportive peers. (Table 1) Adolescent girls in Kenya, Ghana, Botswana, and Uganda had the most parental monitoring (> 70%). Adolescent girls in Kenya, Ghana, Namibia and Swaziland felt the most connected with their parents (> 70%). More than 70% of girls in Botswana, Ghana, Kenya, Namibia, Senegal, and Uganda felt supported by their peers (Table 1).

**Table1:** Characteristics of the social environment of the adolescent girls (N = 22,067)

Country	Parental monitoring		Parental connectedness		Perceived peer support		N	GSHS data year
	Monitored	Not monitored	Connected	Not connected	Supportive	Not supportive		
	N (%)	N (%)	N (%)	N (%)	N (%)	N (%)		
Botswana	811 (70.0)	348 (30.0)	803 (68.2)	375 (31.8)	894 (76.6)	278 (23.4)	1199	2005
Ghana	1979 (70.9)	821 (29.1)	1962 (70.1)	857 (29.9)	2013 (72.6)	770 (27.4)	2917	2007
Kenya	1308 (74.4)	459 (25.6)	1281 (74.1)	469 (25.9)	1348 (74.1)	447 (25.9)	1882	2003
Mauritania	562 (54.1)	476 (45.9)	511 (49.9)	516 (50.1)	597 (57.9)	441 (42.1)	1069	2010
Mozambique	502 (64.0)	334 (36.0)	562 (67.5)	288 (32.5)	509 (56.5)	337 (43.5)	870	2015
Namibia	1,592 (67.5)	743 (32.5)	1677 (72.1)	629 (27.9)	1658 (71.6)	687 (28.4)	2356	2013
Senegal	913 (66.7)	465 (33.3)	805 (60.3)	573 (39.7)	1,012 (72.0)	370 (28.0)	1404	2005
Seychelles	838 (64.1)	458 (35.9)	726 (57)	544 (43)	768 (58.3)	534 (41.7)	1337	2015
Swaziland	2703 (66.9)	1352 (33.1)	2825 (70.4)	1215 (29.6)	2670 (67.0)	1311 (33.0)	4470	2003
Tanzania	1118 (56.5)	806 (43.5)	1030 (52.5)	895 (47.5)	938 (48.1)	963 (51.9)	1935	2014
Uganda	1007 (70.4)	458 (29.6)	1003 (68.6)	481 (31.4)	1060 (72.0)	409 (28.0)	1527	2003
Zambia	589 (63.0)	328 (37.0)	605 (64.6)	320 (35.4)	565 (62.9)	338 (37.1)	1101	2004
All countries	13,922 (64.0)	7048 (36.0)	13,790 (62.1)	7162 (37.9)	14,032 (60.6)	6885 (39.4)	22,067	

The sexual debut prevalence for all adolescent girls was 19.9%. The highest prevalence of sexual debut was observed among adolescent girls in Mozambique (45.8%) and Namibia (43.6%). Adolescent girls in Swaziland had the lowest prevalence (7%) of sexual debut. Among girls who had initiated sex, condom use was more predominant among adolescents’ girls in Botswana (80.8%), Namibia (79.2%) and Mozambique (77.9%). The mean age of sexual debut among all the adolescent girls was 13.21 years and the mean number of sexual partners was 2.19 (Table 2).

**Table 2:** A descriptive results showing the sexual behaviors of adolescent girls in 12 sub-Saharan African countries

Country	Sexual Initiation		Condom Use		Age of sexual debut	Number of sexual partners
	Yes N (%)	No N (%)	Yes N (%)	No N (%)	Mean (95% CI)	Mean (95% CI)
Botswana	160 (17.2)	766 (82.8)	133 (80.8)	32 (19.2)	14.23 (13.75–14.72)	2.08 (1.73–2.43)
Ghana	263 (11.9)	2059 (88.1)	426 (54.7)	368 (45.3)	12.85 (12.67–13.03)	2.63 (2.44–2.83)
Kenya	315 (24.5)	971 (75.5)	147 (44)	182 (56)	12.61 (12.35–12.87)	–
Mauritania	254 (29.3)	618 (70.7)	67 (55.5)	58 (44.5)	12.87 (12.29–13.45)	2.46 (2.05–2.88)
Mozambique	369 (45.8)	385 (54.2)	218 (77.9)	54 (22.1)	14.38 (13.97–14.79)	1.62 (1.46–1.78)
Namibia	849 (43.6)	1185 (56.4)	517 (79.2)	135 (20.8)	14.58 (14.40–14.77)	2.24 (2.10–2.38)
Senegal	129 (11.3)	1014 (88.7)	87 (67.8)	42 (32.2)	13.15 (12.46–13.85)	1.88 (1.48–2.27)
Seychelles	424 (36.9)	769 (63.1)	176 (46.4)	203 (53.6)	13.15 (12.94–13.36)	2.57 (2.35–2.79)
Swaziland	252 (7.0)	3264 (93.0)	121 (48.5)	125 (51.5)	13.09 (12.91–13.26)	2.14 (1.96–2.31)
Tanzania	225 (13.4)	1483 (86.6)	37 (43.5)	48 (56.5)	12.30 (11.80–12.79)	1.94 (1.64–2.24)
Uganda	208 (20.4)	808 (79.6)	129 (62.7)	75 (37.3)	13.82 (13.43–14.22)	2.10 (1.80–2.41)
Zambia	116 (32.6)	250 (67.4)	74 (49.9)	68 (50.1)	12.92 (12.58–13.26)	2.79 (2.45–3.13)
All countries	3564 (19.9)	13,572 (80.1)	2132 (57.5)	1390 (42.5)	13.21 (13.04–13.37)	2.19 (2.08–2.29)

–no data

Results from the independent samples t-test showed significant differences in the mean age of sexual debut and parental attitude, and perceived peer support in four countries, namely, Namibia, Senegal, Seychelles and Tanzania. Significant mean differences in the number of sexual partners were reported in only two countries (17%). In Seychelles and Swaziland parental monitoring and perceived peer support resulted in a significantly lower number of sexual partners respectively.

Overall, in all countries, adolescent girls who felt connected with their parents were older when they initiated sex than those who did not feel connected with their parents (13.34 vs 12.99). In all 12 countries, adolescents who perceived support from their peers had a significantly higher number of sexual partners than those who did not feel supported by their peers (Table 3).

**Table 3:** Mean differences in age of sexual debut and number of sexual partners based on social environment

Country	Social environment		Age of sex debut	T-test (p-value)	Sexual partners	T-test (p-value)
			Mean (95% CI)		Mean (95% CI)	
Botswana	Parental monitoring	Monitored	14.16 (13.66–14.65)	0.426	1.88 (1.37–2.38)	0.197
		Not monitored	14.37 (13.74–14.99)		2.25 (1.86–2.65)	
	Parental connectedness	Connected	14.14 (13.63–14.66)	0.405	1.92 (1.51–2.33)	0.235
		Not connected	14.40 (13.71–15.08)		2.38 (1.66–3.10)	
	Perceived peer support	Supported	14.27 (13.76–14.78)	0.730	2.02 (1.64–2.41)	0.757
		Not supported	14.16 (13.41–14.91)		2.11 (1.46–2.76)	
Ghana	Parental monitoring	Monitored	12.96 (12.73–13.99)	0.145	2.66 (2.37–2.95)	0.753
		Not monitored	12.74 (12.50–12.97)		2.61 (2.41–2.80)	
	Parental connectedness	Connected	12.91 (12.70–13.12)	0.307	2.54 (2.31–2.78)	0.157
		Not connected	12.76 (12.50–13.01)		2.75 (2.51–2.99)	
	Perceived peer support	Supported	12.93 (12.72–13.13)	0.265	2.70 (2.48–2.93)	0.172
		Not supported	12.77 (12.49–13.05)		2.46 (2.16–2.77)	
Kenya	Parental monitoring	Monitored	12.55 (12.22–12.88)	0.579	–	–
		Not monitored	12.73 (12.22–13.24)		–	
	Parental connectedness	Connected	12.61 (12.30–12.91)	0.447	–	–
		Not connected	12.45 (12.09–12.81)		–	
	Perceived peer support	Supported	12.48 (12.23–12.74)	0.306	–	–
		Not supported	12.82 (12.20–13.44)		–	–
Mauritania	Parental monitoring	Monitored	12.83 (12.35–13.31)	0.736	2.66 (2.23–3.09)	0.264
		Not monitored	12.93 (12.04–13.83)		2.23 (1.48–2.98)	
	Parental connectedness	Connected	13.14 (12.62–13.66)	0.235	2.73 (2.27–3.19)	0.093
		Not connected	12.60 (11.64–13.57)		2.21 (1.61–2.81)	
	Perceived peer support	Supported	13.15 (12.54–13.77)	0.10	2.47 (2.02–2.92)	0.417
		Not supported	12.52 (11.68–13.38)		2.33 (1.81–2.85)	
Mozambique	Parental monitoring	Monitored	14.40 (13.79–15.01)	0.673	1.56 (1.39–1.73)	0.289
		Not monitored	14.27 (13.84–14.70)		1.67 (1.47–1.86)	
	Parental connectedness	Connected	14.50 (14.05–14.95)	0.218	1.61 (1.44–1.79)	0.987
		Not connected	14.07 (13.36–14.79)		1.61 (1.41–1.82)	
	Perceived peer support	Supported	14.28 (13.81–14.75)	0.484	1.67 (1.43–1.91)	0.313
		Not supported	14.51 (13.91–15.12)		1.54 (1.39–1.68)	
Namibia	Parental monitoring	Monitored	14.65 (14.45–14.85)	0.186	2.20 (2.06–2.34)	0.470
		Not monitored	14.48 (14.22–14.73)		2.31 (2.03–2.60)	
	Parental connectedness	Connected	14.65 (14.44–14.85)	0.321	2.14 (1.97–2.31)	0.084
		Not connected	14.48 (14.18–14.78)		2.43 (2.16–2.70)	
	Perceived peer support	Supported	14.75 (14.53–14.96)	**0.001**	2.16 (1.99–2.33)	0.108
		Not supported	14.14 (13.86–14.41)		2.46 (2.14–2.79)	
Senegal	Parental monitoring	Monitored	13.08 (12.37–13.79)	0.687	1.68 (1.38–1.99)	0.373
		Not monitored	13.22 (12.29–14.15)		2.15 (1.02–3.28)	
	Parental connectedness	Connected	13.74 (12.74–14.73)	**0.031**	1.66 (1.38–1.94)	0.343
		Not connected	12.70 (12.00–13.39)		2.03 (1.25–2.81)	
	Perceived peer support	Supported	12.99 (12.43–13.55)	0.499	1.78 (1.66–1.90	0.472
		Not supported	13.38 (12.03–14.73)		2.01 (1.11–2.91)	
Seychelles	Parental monitoring	Monitored	13.35 (13.09–13.60	**0.007**	2.35 (2.11–2.59)	**0.01**
		Not monitored	12.90 (12.63–13.16)		2.84 (2.50–3.18)	
	Parental connectedness	Connected	13.16 (12.88–13.43)	0.751	2.42 (2.14–2.71)	0.107
		Not connected	13.11 (12.88–13.34)		2.73 (2.43–3.04)	
	Perceived peer support	Supported	13.22 (12.95–13.49)	0.292	2.50 (2.27–2.73)	0.439
		Not supported	13.06 (12.82–13.30)		2.65 (2.28–3.03)	
Swaziland	Parental monitoring	Monitored	13.12 (12.86–13.39)	0.702	2.13 (1.90–2.37)	0.458
		Not monitored	13.05 (12.77–13.33)		1.97 (1.63–2.31)	
	Parental connectedness	Connected	13.10 (12.86–13.34)	0.672	2.13 (1.83–2.44)	0.945
		Not connected	13.17 (12.93–13.41)		2.11 (1.73–2.50)	
	Perceived peer support	Supported	13.14 (12.92–13.36)	0.465	1.93 (1.76–2.10)	**0.044**
		Not supported	13.00 (12.66–13.34)		2.38 (2.00–2.76)	
Tanzania	Parental monitoring	Monitored	12.93 (12.22–13.65)	**0.013**	1.84 (1.40–2.28)	0.765
		Not monitored	11.97 (11.58–12.36)		1.92 (1.54–2.30)	
	Parental connectedness	Connected	12.31 (11.67–12.94)	0.942	1.94 (1.45–2.44)	1.00
		Not connected	12.28 (11.70–12.87)		1.94 (1.53–2.35)	
	Perceived peer support	Supported	11.88 (11.34–12.42)	**0.039**	1.96 (1.53–2.40)	0.393
		Not supported	12.61 (12.01–13.22)		1.79 (1.51–2.07)	
Uganda	Parental monitoring	Monitored	14.01 (13.56–14.47)	**0.026**	2.16 (1.80–2.51)	0.258
		Not monitored	13.43 (12.99–13.87)		1.92 (1.53–2.30)	
	Parental connectedness	Connected	14.05 (13.51–14.59)	0.070	2.00 (1.61–2.39)	0.350
		Not connected	13.49 (13.06–13.93)		2.22 (1.79–2.64)	
	Perceived peer support	Supported	13.98 (13.44–14.52)	0.139	2.11 (1.75–2.46)	0.941
		Not supported	13.55 (13.26–13.85)		2.13 (1.59–2.67)	
Zambia	Parental monitoring	Monitored	12.88 (12.46–13.31)	0.509	2.79 (2.20–3.38)	0.466
		Not monitored	13.04 (12.49–13.58)		2.48 (1.91–3.04)	
	Parental connectedness	Connected	13.13 (12.71–13.54)	0.182	2.72 (2.28–3.15)	0.982
		Not connected	12.73 (12.11–13.34)		2.72 (2.08–3.36)	
	Perceived peer support	Supported	13.09 (12.62–13.55)	0.152	2.96 (2.34–3.57)	0.225
		Not supported	12.65 (12.08–13.21)		2.38 (1.82–2.95)	
All countries	Parental monitoring	Monitored	13.28 (13.08–13.48)	0.135	2.19 (2.05–2.33)	0.490
		Not monitored	13.10 (12.89–13.31)		2.13 (2.00–2.26)	
	Parental connectedness	Connected	13.34 (13.16–13.52)	**0.002**	2.14 (2.01–2.26)	0.426
		Not connected	12.99 (12.76–13.22)		2.21 (2.06–2.37)	
	Perceived peer support	Supported	13.20 (13.03–13.37)	0.674	2.23 (2.14–2.36)	**0.015**
		Not supported	13.25 (13.00–13.50)		2.03 (1.89–2.17)	

No data; Boldface—significant at < 0.05

Nine Countries (75%) recorded a significant association between parental monitoring and sexual debut. In all nine countries, adolescent girls who were not monitored were more likely to initiate sex than adolescent girls who were monitored. The strongest association was observed in Seychelles (OR = 2.72, 95% CI, 1.29–5.72, p = 0.013). After adjusting for age, class grade and drug use, only three countries (Botswana, Seychelles and Swaziland) observed a significant association between parental monitoring and sexual debut. Overall, in 12 countries, adolescent girls who reported not experiencing monitoring by their parents compared to those who were monitored had 1.29 times the odds to initiate sex. (Table 4).

**Table 4:** Logistic regression model results showing the association between adolescent girls’ social environment and sexual debut

Country	Social environment		Sexual debut			
			OR (95% CI)	p-value	AOR (95% CI)	p-value
Botswana	Parental monitoring	Not monitored	**2.02 (1.37–2.97)**	**0.002**	**1.88 (1.14–3.10)**	**0.017**
		Monitored (ref)				
	Parental connectedness	Not connected	1.30 (0.93–1.83)	0.113	1.12 (0.77–1.62)	0.530
		Connected (ref)				
	Perceived peer support	Not supported	1.12 (0.76–1.65)	0.556	0.99 (0.57–1.72)	0.960
		Supported (ref)				
Ghana	Parental monitoring	Not monitored	**1.55 (1.34–2.12)**	**0.007**	1.15 (0.83–1.60)	0.386
		Monitored (ref)				
	Parental connectedness	Not connected	1.43 (1.00–2.05)	0.052	1.23 (0.77–1.96)	0.379
		Connected (ref)				
	Perceived peer support	Not supported	**1.54 (1.14–2.08)**	**0.006**	1.22 (0.83–1.79)	0.309
		Supported (ref)				
Kenya	Parental monitoring	Not monitored	0.98 (0.54–1.68)	0.948	0.84 (0.41–1.74)	0.628
		Monitored (ref)				
	Parental connectedness	Not connected	1.12 (0.80–1.58)	0.506	1.15 (0.76–1.73)	0.502
		Connected (ref)				
	Perceived peer support	Not supported	**1.38 (1.001–1.90)**	0.049**	1.33 (0.98–1.79)	0.063
		Supported (ref)				
Mauritania	Parental monitoring	Not monitored	1.13 (0.69–1.85)	0.612	1.26(0.73–2.18)	0.379
		Monitored (ref)				
	Parental connectedness	Not connected	1.01 (0.58–1.74)	0.981	0.72 (0.4–1.29)	0.244
		Connected (ref)				
	Perceived peer support	Not supported	1.48 (0.85–2.56)	0.147	1.35 (0.7–2.62)	0.341
		Supported (ref)				
Mozambique	Parental monitoring	Not monitored	**1.43 (1.07–1.89)**	**0.018**	1.28 (0.82–1.99)	0.256
		Monitored (ref)				
	Parental connectedness	Not connected	1.19 (0.81–1.75)	0.360	0.99 (0.68–1.43)	0.936
		Connected (ref)				
	Perceived peer support	Not supported	1.04 (0.59–1.82)	0.886	0.98 (0.60–1.61)	0.943
		Supported (ref)				
Namibia	Parental monitoring	Not monitored	**1.49 (1.12–1.99)**	**0.009**	1.24 (0.91–1.71)	0.171
		Monitored (ref)				
	Parental connectedness	Not connected	**1.43 (1.05–1.93)**	**0.024**	1.34 (0.96–1.88)	0.083
		Connected (ref)				
	Perceived peer support	Not supported	1.21 (0.95–1.55)	0.116	1.25 (0.97–1.62)	0.076
		Supported (ref)				
Senegal	Parental monitoring	Not monitored	1.76 (0.92–3.38)	0.084	1.09 (0.59–2.01)	0.770
		Monitored (ref)				
	Parental connectedness	Not connected	**2.72 (1.29–5.72)**	**0.013**	2.36 (0.98–5.68)	0.054
		Connected (ref)				
	Perceived peer support	Not supported	**2.36 (1.31–4.25)**	**0.008**	**1.73 (1.19–2.53)**	**0.008**
		Supported (ref)				
Seychelles	Parental monitoring	Not monitored	**2.30 (1.71–3.10)**	**0.000**	**2.27 (1.61–3.20)**	**0.000**
		Monitored (ref)				
	Parental connectedness	Not connected	**1.53 (1.19–1.96)**	**0.001**	1.04 (0.76–1.40)	0.818
		Connected (ref)				
	Perceived peer support	Not supported	1.21 (0.93–1.57)	0.150	1.10 (0.84–1.44)	0.485
		Supported (ref)				
Swaziland	Parental monitoring	Not monitored	**1.75 (1.33–2.30)**	**0.000**	**1.59 (1.17–2.15)**	**0.003**
		Monitored (ref)				
	Parental connectedness	Not connected	**1.93 (1.48–2.51)**	**0.000**	1.35 (0.94–1.94)	0.102
		Connected (ref)				
	Perceived peer support	Not supported	1.20 (0.89–1.62)	0.229	0.99 (0.69–1.41)	0.949
		Supported (ref)				
Tanzania	Parental monitoring	Not monitored	**1.50 (1.09–2.06)**	**0.014**	1.01 (0.69–1.48)	0.946
		Monitored (ref)				
	Parental connectedness	Not connected	**1.90 (1.43–2.53)**	**0.000**	**1.78 (1.25–2.53)**	**0.003**
		Connected (ref)				
	Perceived peer support	Not supported	1.35 (0.99–1.83)	0.761	1.23 (0.94–1.60)	0.120
		Supported (ref)				
Uganda	Parental monitoring	Not monitored	**1.60 (1.02–2.53)**	**0.044**	1.41 (0.74–2.68)	0.278
		Monitored (ref)				
	Parental connectedness	Not connected	**1.95 (1.44–2.63)**	**0.000**	**1.92 (1.32–2.78)**	**0.001**
		Connected (ref)				
	Perceived peer support	Not supported	1.38 (0.94–2.04)	0.098	1.04 (0.75–1.44)	0.796
		Supported (ref)				
Zambia	Parental monitoring	Not monitored	**2.03 (1.01–4.09)**	**0.047**	0.92 (0.39–2.17)	0.832
		Monitored (ref)				
	Parental connectedness	Not connected	**2.06 (1.18–5.71)**	**0.021**	2.12 (0.87–5.12)	0.092
		Connected (ref)				
	Perceived peer support	Not supported	0.92 (0.50–1.67)	0.761	0.81 (0.38–1.74)	0.563
		Supported (ref)				
All countries	Parental monitoring	Not monitored	**1.29 (1.09–1.52)**	**0.003**	1.12 (0.93–1.35)	0.231
		Monitored (ref)				
	Parental connectedness	Not connected	**1.30 (1.13–1.51)**	**0.000**	**1.32 (1.14–1.53)**	**0.000**
		Connected (ref)				
	Perceived peer support	Not supported	1.16 (0.99–1.37)	0.075	1.13 (0.97–1.31)	0.119
		Supported (ref)				

–adjusted for age, class Grade and drug use; **Borderline significance

Seven out of the twelve countries (58%) observed a significant association between parental connectedness and sexual debut. Adolescent girls in Senegal reported the strongest association between lack of parental connectedness and sexual debut (OR = 1.32, 95% CI, 1.14–1.53, p < 0.000) (OR = 2.72). Of the seven countries that observed a significant association, two countries ( Tanzania, and Uganda) remained significant after adjusting for age, class grade and drug use. The results from the pooled data of all 12 countries showed that adolescents who were not connected with their parents were 1.32 times as likely to initiate sex compared to adolescent girls who felt connected to their parents. (Table 4).

With respect to perceived peer support, only three countries (25%) observed a significant association between perceived peer support and sexual debut. Adolescent girls who did not feel supported by their peers were more likely to initiate sex in Ghana, Kenya and Senegal. This association remained significant in Senegal (aOR = 1.73, 95% CI, 1.19–2.53, p = 0.008) after controlling for age, class grade and drug use (Table 4).

## Discussion

The study findings indicate that adolescent girls in sub-Saharan Africa whose parents did not know what they did during their free time (not monitored by their parents) were more likely to initiate sex compared to their counterparts who were monitored by their parents. Likewise, adolescents’ girls who felt their parents or guardians did not understand their problems and worries (not connected with their parents) were also more likely to initiate sex compared to adolescent girls who felt connected with their parents.

Our findings are consistent with previous studies conducted in some countries in sub-Saharan Africa. In Ghana, Kumi-Kyereme et al. [16] found that adolescent girls who were monitored by their parents were less likely to be sexually active. Dimbuene and Defo [14] also conducted a study on family environment and premarital intercourse in Cameroon, and found that parents who had a stronger relationship with their children and exercised higher levels of parental control helped reduce the risk of premarital intercourse. In the United States, Diclemente et al. [17] found that adolescents who perceived less parental monitoring were more likely to test positive for a sexually transmitted disease, not use condoms at the last sexual intercourse and also to have multiple sexual partners.

The study results showed that adolescent girls in all 12 countries, who felt supported by their peers, had a significantly higher number of sexual partners. Previous studies have also found peer influence on adolescent sexual behaviors [18, 19, 23].

We found that the age of sexual debut differed among adolescents in the countries included in our study. This is consistent with previous studies. For example, Melesse et al. [29] found that girls in West and Central Africa reported a younger age of sexual debut. Studies indicate that adolescent girls who initiate sex early are less likely to use condoms and other contraceptives at first sex, thereby increasing their risk of pregnancy and sexually transmitted infections (STIs) [30–32]. Also, according to Magnusson and Trost [33] early sexual activity in girls has also been associated with higher levels of gynecological problems. Early initiation of sex could result in teenage pregnancy which according to Yakubu and Salisu [34] may contribute to denying students access to education and potentially affect their growth and the development of their children.

The study utilized the conceptual model of the relationship between social networks and social support on health proposed by Heaney and Israel [24]. The study results support this conceptual model that social networks and social support (parental monitoring, parental connectedness, and peer support) may influence behavioral risk factors such as early sexual debut, age of sexual debut and number of sexual partners. We suggest that health policies targeting the sexual behaviors of adolescent girls in SSA should prioritize involving the family especially parents and caregivers and also peer support to reduce teenage pregnancies and STI.

### Strengths and limitations

The study is one of the few studies to explore the association between the social environment of adolescent girls and their sexual behaviors in sub-Saharan Africa. Despite this strength, the study had a few limitations. The GSHS data surveys school going adolescents as such adolescent girls who might have dropped out of school may not have been captured.

Secondly, adolescents had to recall their sexual behaviors during the past 30 days. This approach could lead to the potential of recall bias. Also, the GSHS questions were used as proxy for this study, thus the parental influence scale and the perceived peer support scale tailored towards sexual behaviors among adolescents would have been preferred. For example, previous research has shown that self-efficacy, presence of sexual coercion, age of partner, partner-type could influence adolescent sexual behaviors [35, 36] but these variables are not available in the GSHS data. Therefore, the study warrants caution with interpretation.

The secondary nature of our data analysis limits the exploration of additional relevant variables, and by extent, the measurement protocols utilized by the CDC and the WHO. We do note that these variables are reliable and have been used in other published studies [37, 38]. This was also a cross-sectional study, so we did not follow the adolescent girl’s trajectory from their social environment to their sexual behaviors.

We conveniently sampled countries that GSHS national data that was publicly available, and the data years ranged from 2003 to 2015. We acknowledge that they might be some behavioral changes among adolescent girls in the countries included in the study since the GSHS data was collected. Thus, the study warrants caution with interpretation.Lastly, we would have preferred to also examine whether differences exist in adolescent girls’ sexual debut among girls living in single parent homes compared to those living with both parents, but the data was not available in the GSHS survey.

## Conclusion

Adolescent girls who are not monitored and do not feel connected with their parents are more likely to debut sex early. We conclude that adolescent girls' social environment in sub-Saharan Africa plays a vital role in sexual debut, age of sexual debut, and the number of sexual partners.

## Data Availability

The study used the Global School-based Student Health Survey (GSHS) data which is a publicly available data. Available from: http://www.who.int/chp/gshs/datasets/en/.

## References

[1] World Health Organization. Global health risks: mortality and burden of disease attributable to selected major risks. Geneva; 2009.

[2] UN Department of Social and Economic Affairs PD. World population prospects: highlights of the 2008 revision. Working Paper No ESA/P/WP.210. New York, NY: United Nations.; 2009.

[3] Ringheim K, Gribble J. Improving the reproductive health of sub-Saharan Africa’s youth, a route to achieve the millennium development goals. Population Reference Bureau. Washington, DC; 2010.

[4] UNICEF. Child poverty in perspective: an overview of child well-being in rich countries, Innocenti Report Card 7. 2007.

[5] World Health Organization. Adolescent friendly health services : an agenda for change. World Health Organization. 2003. https://apps.who.int/iris/handle/10665/67923.

[6] Sawyer SM, Afifi RA, Bearinger LH (2012). Adolescence: a foundation for future health. Lancet.

[7] World Health Organization. Health for the World’s Adolescents. A second chance in the second decade. Geneva; 2014.

[8] Singh S, Darroch J E, Ashford LS, Vlassoff M. Adding it up: The Costs and Benefits of Investing in Family Planning and Maternal and Newborn Health, New York: Guttmacher Institute and United Nations Population Fund, 2009.

[9] Beguy D, Mumah J, Gottschalk L (2014). Unintended pregnancies among young women living urban slums evid from a prospect study Nairobi City, Kenya. PLoS ONE.

[10] Clark S (2004). Early marriage and HIV risks in sub-Saharan Africa. Stud Fam Plann.

[11] Cleland J, Boerma JT, Carael M, Weir SS (2004). Monitoring sexual behaviour in general populations: a synthesis of lessons of the past decade. Sex Transm Infect..

[12] United Nations Department of Economic and Social Affairs. World Fertility Patterns 2015—Data Booklet (ST/ESA/ SER.A/370). 2015.

[13] Ganchimeg T, Ota E, Morisaki N (2014). Pregnancy and childbirth outcomes among adolescent mothers: a World Health Organization multicountry study. BJOG.

[14] Dimbuene TZ, Defo KB (2012). Family environment and premarital intercourse in Bandjoun (West Cameroon). Arch Sex Behav.

[15] Ngom P, Magadi MA, Owuor T (2003). Parental presence and adolescent reproductive health among the Nairobi urban poor. J Adolesc Heal.

[16] Kumi-Kyereme A, Awusabo-Asare K, Biddlecom A, Augustine T (2007). Influence of social connectedness, communication and monitoring on adolescent sexual activity in Ghana. Afr J Reprod Heal.

[17] DiClemente RJ, Wingood GM, Crosby RA (2001). A prospective study of psychological distress and sexual risk behavior among black adolescent females. Pediatrics.

[18] Connolly J, Furman W, Konarski R (2000). The role of peers in the emergence of heterosexual romantic relationships in adolescence. Child Dev.

[19] Sieving RE, Eisenberg ME, Pettingell S, Skay C (2006). Friends’ influence on adolescents’ first sexual intercourse. Perspect Sex Reprod Heal.

[20] Kinsman SB, Romer D, Furstenberg FF, Schwarz DF (1998). Early sexual initiation: the role of peer norms. Pediatrics.

[21] Carvajal SC, Parcel GS, Banspach SW (1999). Psychosocial predictors of delay of first sexual intercourse by adolescents. Health Psychol.

[22] Vanoss Marín B, Coyle KK, Gómez CA (2000). Older boyfriends and girlfriends increase risk of sexual initiation in young adolescents. J Adolesc Health.

[23] Adongo B (2018). Assessing factors influencing early sexual initiation among adolescents (13 to 19 years ) in Ghana. Int J Caring Sci.

[24] Heaney CA, Israel BA. Social networks and social support. In Glanz K, Rimer BK, Viswanath K, editors, Health behavior and health education: Theory, research, and practice, 2008, pp 189–210.

[25] Centers for Disease Control and Prevention, World Health Organization. Global School-Based Health Survey. http://www.cdc.gov/gshs/pdf/GSHSOVerview.pdf.

[26] World Health Organization. Noncommunicable diseases and their risk factors. Global school-based student health survey (GSHS) . http://www.who.int/chp/gshs/datasets/en/.

[27] Global School-Based Student Health Survey (GSHS). CoreQuestionnaire Modules. http://www.who.int/chp/gshs/GSHS_Core_Modules_2009_English.pdf.

[28] Centers for Disease Control and Prevention, World Health Organization. Global Student Health Survey (GSHS) Data User’s Guide. 2013. http://www.who.int/chp/gshs/methodology/en/index.htmlhttp://www.cdc.gov/gshs/questionnaire/index.htm.

[29] Melesse DY, Mutua MK, Choudhury A (2020). Adolescent sexual and reproductive health in sub-Saharan Africa: who is left behind?. BMJ Glob Heal.

[30] Heywood W, Patrick K, Smith AM, Pitts MK (2015). Associations between early first sexual intercourse and later sexual and reproductive outcomes: a systematic review of population-based data. Arch Sex Behav.

[31] Langille DB, Asbridge M, Flowerdew G, Allen M (2010). Associations of sexual risk-taking with having intercourse before 15 years in adolescent females in Cape Breton, Nova Scotia, Canada. Sex Health.

[32] Olesen TB, Jensen KE, Nygård M (2012). Young age at first intercourse and risk-taking behaviours—a study of nearly 65 000 women in four Nordic countries. Eur J Public Health.

[33] Magnusson C, Trost K (2006). Girls experiencing sexual intercourse early: could it play a part in reproductive health in middle adulthood?. J Psychosom Obstet Gynecol.

[34] Yakubu I, Salisu W (2018). Determinants of adolescent pregnancy in sub-Saharan Africa: a systematic review. Reprod Health..

[35] Moore AM, Awusabo-Asare K, Madise N (2007). Coerced first sex among adolescent girls in sub-Saharan Africa: prevalence and context. Afr J Reprod Health.

[36] Ybarra, ML, Mitchell KJ, Espelage DL. Comparisons of bully and unwanted sexual experiences online and offline among a national sample of youth. In: Özdemir Ö, editor. Complementary Pediatrics. InTech; 2012. p. 204–16. 10.5772/33532.

[37] Darfour-Oduro SA, Andrade J, Grigsby-Toussaint DS (2020). Do Fruit and vegetable policies, socio-environmental factors, and physical activity influence fruit and vegetable intake among adolescents?. J Adolesc Health.

[38] Peltzer K, Pengpid S (2010). Fruits and vegetables consumption and associated factors among in-school adolescents in seven African countries. Int J Public Health.

